# Patient access to orphan drugs in France

**DOI:** 10.1186/s13023-019-1026-4

**Published:** 2019-02-18

**Authors:** Marion Bourdoncle, Blandine Juillard-Condat, Florence Taboulet

**Affiliations:** 1 Department of pharmacy, CHU de Toulouse, 293 chemin de Tucaut, 31270 Cugnaux, France; 2 INSERM UMR 1027, Toulouse, 37 allées Jules Guesde, 31000 Toulouse, France; 3 University of Toulouse III, Toulouse, France

**Keywords:** Orphan drugs, Rare disease, Patient access, Availability

## Abstract

**Background:**

Since incentives were introduced to promote orphan drugs in Europe, several dozens of drugs have been registered at the European level. However, patient access on a national level remains very heterogeneous across Europe. This can be explained by healthcare organization and drug reimbursement, which are within the purview of each Member State. We studied access to orphan drugs in France from the patients’ point of view, including marketing but also ease of supply from patients’ perspective, financial and time-based dimensions.

**Results:**

We identified 91 registered orphan drugs in Europe, corresponding to 115 orphan drug–therapeutic indication pairs. In France, 78.3% (90/115) of these pairs were marketed: 100% were available to inpatients and 75.6% were available to outpatients. The median period between granting of the European marketing authorization and publication of the reimbursement decision was 360 days. The broadest availability—through community pharmacies—was guaranteed in only 31.1% of cases. Prescriptions were mainly restricted either to hospital-based doctors or to specialists. Inpatients were not financially responsible for these prescriptions and 72% of the orphan drug–therapeutic indication pairs available to outpatients were fully covered by national health insurance in France.

**Conclusions:**

Patient access to orphan drugs is not universal in France. Access to reimbursement has a strong impact on patients’ effective access to orphan drugs, which may be restricted by difficulties with assessing the clinical value of these drugs and with pricing issues. Prescribing restrictions and drug delivery systems influence the ease of patients’ supply for reimbursed orphan drugs for patients. Patients do not seem to be limited by financial issues, but the growing budgetary impact of orphan drugs is worrisome from a societal point of view.

## Introduction

Rare diseases affect around 30 million people in the European Union (EU) and many of them are life-threatening [1]. To encourage the development of orphan drugs, the EU implemented a specific policy— Regulation (EC) No. 141/2000—in 2000. Largely inspired by the US Orphan Drug Act, it provides a set of incentives to promote the development and marketing of drugs for serious and rare diseases lacking a satisfactory therapeutic alternative. Drugs are designated as orphan drugs during the development process. Then, they are evaluated to obtain European marketing authorization (MA). Since 2000, several dozen orphan drugs have been registered through a centralized procedure.

In 2010, the European Organization for Rare Diseases (EURORDIS) studied the market access of 60 approved orphan drugs (i.e. drugs with an orphan designation and a MA) in 10 EU countries. In France, 90% of these drugs were available but this proportion was reduced to 33% in some of the countries studied [2]. The results of EURORDIS study also show that orphan drugs for the rarest diseases were the least accessible, and that accessibility was not just a question of time: for each orphan drug, there was no progress in availability across EU after the first 2 years following MA. In spite of a common European desire to promote orphan drugs, national access for patients is still very heterogeneous across Europe [3] and represents a real public health issue. MA ensures regulatory market access but is not synonymous with effective access. To evaluate patients’ effective access to orphan drugs, four dimensions must be considered: availability of the drug, ease of supply from patients’ point of view, level of reimbursement and delay between MA and effective access to the drug. As healthcare organization and drug reimbursement by a national health insurance scheme are within the purview of each Member State, these four dimensions should be analyzed at national policy level. In this context, an assessment of the four dimensions determining effective access to orphan drugs after MA was conducted in France. All extractions were performed in August 2016.

**Fig. 1:**
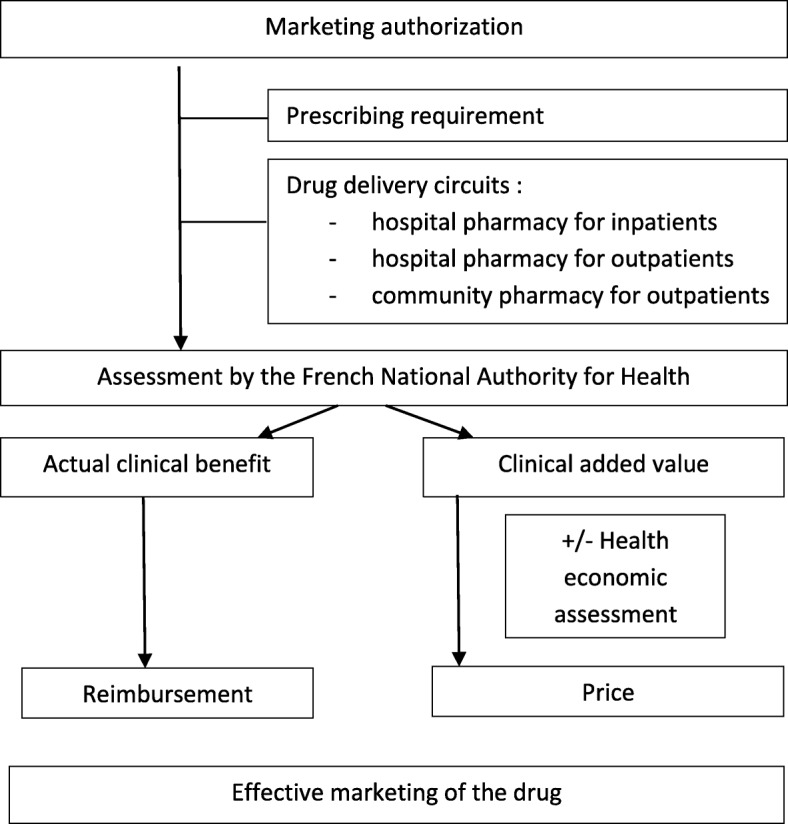
Synthetic diagram of the market access in France

In France, National Plans for Rare Diseases promote equal access to diagnosis and care throughout the country [4]. In particular, expert physician networks are responsible for ensuring appropriate use of orphan drugs. After MA, reimbursement decisions for each therapeutic indication of orphan and non-orphan drugs are based on the opinion of the French National Authority for Health (*Haute Autorité de Santé* - HAS). Mandatory criteria for this health technology assessment are actual clinical benefit and clinical added value. The actual clinical benefit is rated on a 4-level scale, and takes into account 5 criteria: severity of the disease/condition, efficacy, adverse effects, intended place in the therapeutic strategy in comparison with other available therapies, and public health benefits. For indications with insufficient actual clinical benefit, the HAS gives a negative opinion for inscription on the list of reimbursed drugs. The final decision of reimbursement is the responsibility of the Ministry of Health. The clinical added value is rated on a 5-level scale, and evaluates comparative efficacy and safety data with regards to available treatments (reference medicinal product or better treatment modalities). In addition, a health economic assessment may be required. Both clinical added value and health economic assessment are criteria used during price negotiation with the pharmaceutical companies. Prescribing and/or dispensing restrictions may limit drug access for safety reasons. Three drug delivery circuits exist: 1) drug dispensing by community pharmacies to outpatients, 2) drug dispensing by hospital pharmacies to inpatients and 3) drug dispensing by hospital pharmacies to outpatients (commonly named “retrocession*”* in France) [5]. Funding mechanisms and rate of reimbursement vary according to drug delivery circuits and HAS opinions. Figure 1 presents a synthetic diagram of French market access for drugs.

In previous studies, accessibility was measured either by market access [2] or by uninterrupted sales within a 1-year period [3], without specifically identifying the impact of reimbursement and healthcare organization on patient access to orphan drugs. In this study, the assessment of effective access of French patients to orphan drugs is based on four factors: marketing, ease of supply, financial and time-based dimensions. Patients’ perspective is adopted for ease of supply and financial dimension.

## Material and methods

The study was performed in August 2016. A list of active “orphan drug–therapeutic indication” pairs was extracted from the community register of orphan drugs [6]. We focused on drugs with both an orphan designation and a MA for the orphan condition. Patient access to orphan drugs was assessed through 4 dimensions:

- effective marketing of the drug, that means availability of the drug on the French market;
- ease of supply from patient perspective, assuming that some prescribing or dispensing restrictions may be barriers to accessibility;
- financial access (funding and rate of reimbursement);
- access time (between MA and publications of decisions relatives to reimbursement and price).

Information on HAS assessments, prescribing restrictions and drug delivery circuits were collected from several French websites and databases: National Drug Safety Agency [7], French National Authority for Health [8], and National Health Insurance [9].

In order to prevent delays in drug access, compassionate use of an orphan drug (through the French temporary licensing system – temporary authorization for use, ATU) is possible before the MA is granted, but was not taken into account in our study. Once the MA is obtained, drugs that previously received an ATU can benefit from a transitional provision (so called post-ATU) that continues to provide access after the MA until the ministerial decision on reimbursement and pricing has been made. During the post-ATU period, access is granted both for inpatients and outpatients, but only from hospital pharmacies. Orphan drugs in the post-ATU period were included in our study.

## Results

Our study identified 91 orphan drugs, corresponding to 115 orphan drug–therapeutic indication pairs; 16.5% (15/91) of identified drugs were registered in more than one designated orphan condition. Results are summarized in Table 1.

**Table 1:** Orphan drugs in France in August 2016

	Number	Percent
Active MA and orphan designation		
Number of drugs	91	–
Number of active designated orphan indications by drug		
- 1 rare disease	76 / 91	83.5
- 2 rare diseases	10 / 91	11.0
- 3 rare diseases	3 / 91	3.3
- 4 rare diseases	1 / 91	1.1
- 6 rare diseases	1 / 91	1.1
Total number of orphan drug–therapeutic indication pairs	115	–
Assessment of orphan drug–therapeutic indication pairs (*N* = 115) by the HAS		
Number of assessed orphan drug–therapeutic indication pairs (i.e. published HAS opinions)	94/115	81.7
Not found on the HAS website	18/115	15.7
Assessment is on-going	2/115	1.74
Withdrawn by pharmaceutical company	1/115	0.90
Actual clinical benefit of published HAS opinions		
- Important	72/94	76.6
- Moderate	17/94	18.1
- Mild	1/94	1.1
- Insufficient (not accepted for reimbursement)	4/94	4.3
Clinical Added Value of published HAS opinions		
- Major	2/94	2.1
- Important	16/94	17.0
- Moderate	20/94	21.3
- Minor	36/94	38.3
- No clinical improvement	16/94	17.0
- Not applicable (because of insufficient actual clinical benefit)	4/94	4.3
Eligibility for health economic assessment	12/115	-^a^
Adopted efficiency opinions	3/12	25.0
- with major methodological criticisms expressed by the HAS	2/3	66.7
Published efficiency opinion	1/3	33.3
Marketing of registered orphan drug–therapeutic indication pairs		
Number of marketed orphan drug–therapeutic indication pairs	90/115	78.3
- through the post-ATU transitional provision	15/90	16.7
Number of non-marketed orphan drug–therapeutic indication pairs	25/115	21.7
Regulatory prescribing requirements of marketed orphan drug–therapeutic indication pairs (*N* = 90)		
Reserved for hospital use	19 / 90	21.1
On hospital prescription	56 / 90	62.2
On initial hospital prescription	14 / 90	15.6
On specialist doctor’s prescription	60 / 90	66.7
Specific monitoring	55 /90	61.1
Drug delivery circuits for marketed orphan drug–therapeutic indication pairs (N = 90)		
Hospital pharmacies for inpatients	90/90	100
- through the post-ATU transitional provision	15/90	16.7
Community pharmacies	28/90	31.1
Hospital pharmacies for outpatients (retrocession)	40/90	44.4
- through the post-ATU transitional provision	13/40	32.5
Funding / theoretical reimbursement ratesss		
Inpatients (hospital)	-	-
- universal reimbursement (in connection with Diagnosis Related Groups)	52/90	57.8
- high-priced drug list	23/90	25.5
- specific to the post-ATU transitional provision	15/90	16.7
Outpatients (hospital pharmacies) = retrocession	-	-
- full reimbursement (100%)	33/40 including 13 post-ATU	82.5
- reimbursement up to 65%	6/40	15.0
- reimbursement up to 30%		2.5
	1/40	
Community pharmacies	-	-
- full reimbursement (100%)	16/28	57.1
- reimbursement up to 65%	9/28	32.1
- reimbursement up to 30%	3/28	10.7

^a^Not applicable because health economic evaluations were set up in France in October 2012 [53]

### Evaluation by the French National Authority for health (HAS)

At the time of the study, a published HAS opinion was available for 94/115 (81.7%) identified orphan drug–therapeutic indication pairs. Among the 94 pairs with a published HAS opinion, only 4 had a negative opinion for reimbursement, based on an insufficient actual clinical benefit. A major or important clinical added value was recognized to 18/94 (19.1%) pairs. The median period between the granting of the MA and the adoption of the HAS opinion was 227 days. An additional health economic assessment was required for 12 orphan drug–therapeutic indication pairs.

### Market access

Overall, 78.3% (90/115) of the orphan drug–therapeutic indication pairs approved in Europe were available to patients in France. The median period between the granting of the European MA and French access to the reimbursed drug market (i.e. publication of ministerial decisions) was 360 days.

Compassionate use (i.e. ATU) had provided access before the MA for 55/115 of the studied orphan drug–therapeutic indication pairs. At the time of our study, 15/90 (16.7%) orphan drug–therapeutic indication pairs were available through the post-ATU transitional provision.

**Fig. 2:**
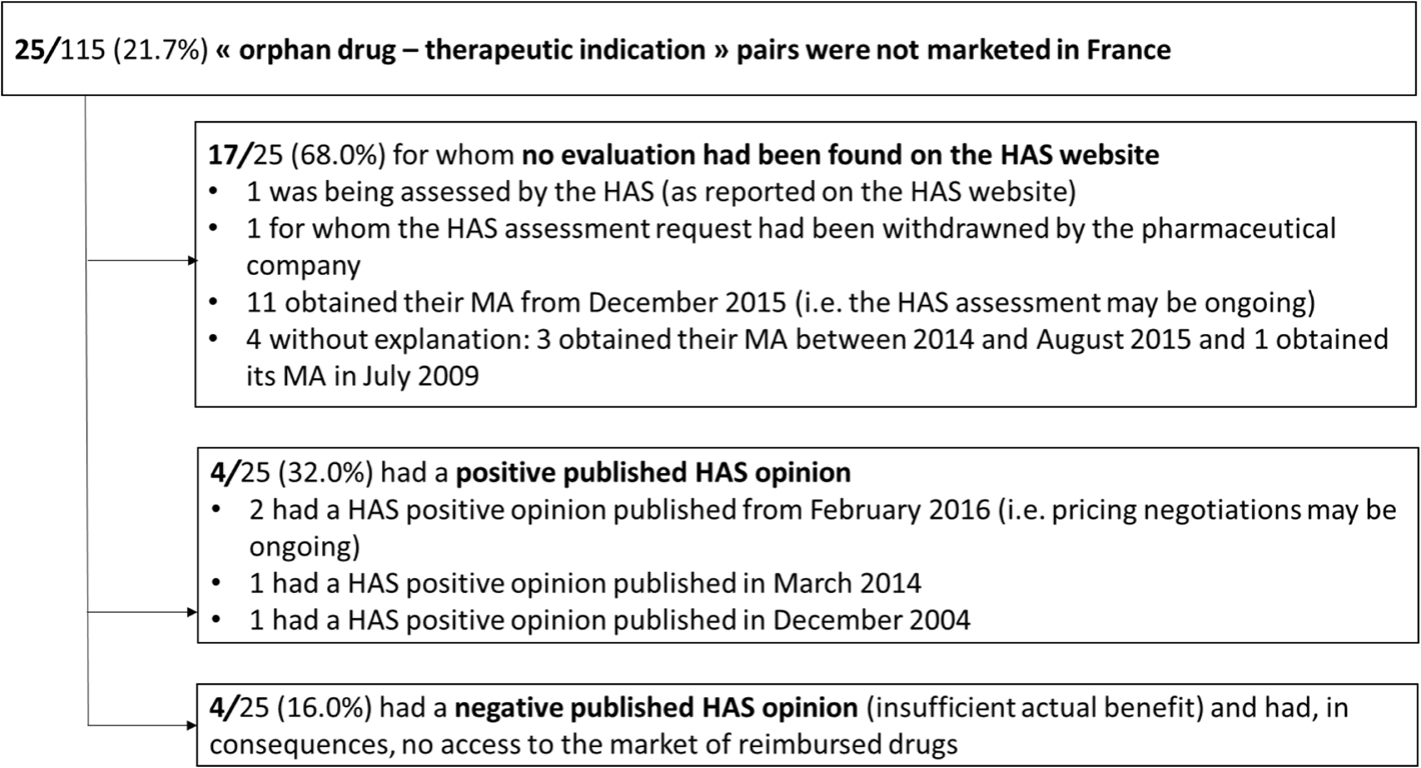
Assessment of “orphan drug - therapeutic indication” pairs not marketed in France

Concerning the 25/115 (21.7%) orphan drug–therapeutic indication pairs with active MA and orphan designation that were not marketed in France (Fig. 2):

- 17/25 (68.0%) had no evaluation available on the HAS website. The reasons identified for the lack of HAS opinion was: assessment in progress as reported on the HAS website (1/17), withdrawal of pharmaceutical company request (1/17), assessment may be ongoing at the time of the study (11/17) and no explanation for the remaining 4/17;
- 4/25 (16%) were not included in the list of reimbursed medicines at the time of the study despite HAS positive opinions published respectively in December 2004 [1], March 2014 [1] and February 2016 [2]. For the last two, pricing negotiation may be ongoing at the time of the study;
- 4/25 (16%) were negatively evaluated by the HAS, and none of them was included in the list of reimbursed medicines.

These results allow to hypothesize that the lack of availability of an orphan drug on French market can be explained by:

- factors related to health technology assessment delays (14/25) or results (4/25), representing globally 72% (18/25) of cases;
- factors probably related to industrial strategies (7/25) for 28% of cases.

### Prescribing restrictions

The use of orphan drug–therapeutic indication pairs was regulated: 21.1% (19/90) were reserved for hospital use, 61.1% (55/90) required specific monitoring, 62.2% (56/90) were available only on hospital prescription, 15.6% (14/90) were available only on initial hospital prescription. Prescriptions were restricted to some specialist doctors for 66.7% (60/90) of them.

### Access facility from patients’ perspective

Orphan drugs were mostly managed in hospitals: 68.9% (62/90) were purchased and delivered by hospital pharmacies to inpatients and outpatients. The retrocession mechanism provided outpatients with access to 44.4% (40/90) of orphan drug-therapeutic indication pairs. Inpatients had access to 100% (90/90) and outpatients had access to 75.6% (68/90) of orphan drug–therapeutic indication pairs. The broadest access (i.e. through community pharmacies) was guaranteed in 31.1% (28/90) of cases.

### Financial access

No financial contribution for orphan drugs was requested for inpatients. For outpatients (through both the retrocession circuit and community pharmacies), 72% (49/68) of orphan drug–therapeutic indication pairs were fully reimbursed. However, 22.1% (15/68) of the orphan drug–therapeutic indication pairs were reimbursed up to 65 and 5.9% (4/68) of them up to 30%.

## Discussion

Orphan drug status has encouraged pharmaceutical investment and hence market access for treating rare and serious diseases [10]. In this manner, the European policy on orphan drugs has fulfilled its objective by making these diseases more attractive from a commercial point of view. However, the main purpose of this policy is to address highly unmet medical needs. The objective of our study was to assess the impact of this European policy by measuring the effective access to orphan drugs from the patients’ point of view in France. Our results show that 78.3% of approved orphan drug–therapeutic indication pairs were marketed, among which only 31.1% had the broadest availability and 72% were fully reimbursed for outpatients. Our study was limited to France because of large differences between Member States’ policies and health technology assessments [11].

As we focused on registered orphan drugs, our study underestimates the real access to drugs for rare diseases in France. First, drugs registered in rare diseases but that were not designated as orphan drugs were excluded. Then we also excluded drugs with an orphan designation for which the MA application was still in progress, even if they were available through the compassionate use provision (i.e. ATU), which is known to accelerate drug access for patients [12]. Lastly, drugs already marketed for other conditions and available for rare diseases through the temporary recommendation for use *(RTU)* provision were not taken into account. While these drugs can be used for off-label indications, their use is accompanied by strict data collection requirements. The final goal of this RTU provision is to expand the approved therapeutic indications.

At the time of our study, 78.3% of the registered orphan drug–therapeutic indication pairs were marketed in France. In a survey conducted and published by EURORDIS in 2010 [2], 90% of the studied orphan drugs were available in France. Several factors can explain this difference: 1) our study is more recent and more exhaustive; 2) we studied orphan drug–therapeutic indication pairs because reimbursement is granted by therapeutic indication and we hypothesized that reimbursement has a big impact on marketing. A total of 16.5% of identified orphan drugs were indicated in at least two orphan conditions. However, there is a bias between our theoretical approach and reality: when reimbursement is given for one therapeutic indication, the marketed drug can be used in another non-reimbursed indication based on medical argument for hospital delivery circuits or because validation of the indication is not always required for the community delivery circuit.

For marketed orphan drugs, the median delays measured between MA and HAS opinion, and between MA and ministerial decisions of reimbursement and pricing were consistent with delays reported in another French study conducted on a sample of a hundred of orphan and non-orphan drugs [13]: respectively 227 days versus 184 days between MA and HAS opinion, and 360 days versus 359.5 days between MA and decisions of reimbursement and pricing. The negotiation phase for orphan drug prices could be shorter for two reasons. First, the high clinical added value ensures the price is consistent with European prices. The second reason is the specific French provision of “capping” [14]. For orphan drugs with an annual cost per patient exceeding €50,000, “in return for the acceptance of a price consistent with international prices, [the pharmaceutical company undertakes] to supply the drug to all patients eligible for treatment, without any restriction, for a total amount of revenue fixed in advance”. So access to reimbursement and pricing did not appear to be slowed by the difficulties inherent to the scarcity of therapeutic indications in France.

Table 1 results demonstrate that in France, hospital capabilities are needed for the majority of marketed orphan drugs. These prescribing and dispensing requirements promote the proper use of orphan drugs and careful patient monitoring, while helping regulate healthcare expenditures [15]. It can be noticed orphan drugs are four times more likely to be provided through retrocession than all the brand-name drugs marketed in France (i.e. orphan and non-orphan drugs): retrocession concerns 44.4% of orphan drugs versus 10.8% of all drugs marketed in France [16, 17]. This restrictive distribution circuit allows special monitoring of prescriptions and deliveries [18]. Yet better safety should be balanced with restrictions of patients accessibility: according to 2017 statistics of the French chamber of pharmacists, there are 21,815 community pharmacies in France, and only 2445 hospital pharmacies [19]. It should be noticed that 32.5% (13/40) of orphan drug–therapeutic indication pairs subject to retrocession were available through the post-ATU provision. At the end of this transitional period, some of them may be delivered by community pharmacies.

All of the orphan drug–therapeutic indication pairs available to inpatients and 72% of the pairs available to outpatients were fully covered by the national health insurance scheme in France. For inpatients, funding was either universal (in connection with Diagnosis related groups), or related to extra funding granted to hospitals for high-cost drugs in return for appropriate use, or specific to the post-ATU transitional provision. For outpatients, when there was a theoretical excess to be paid by patients, the actual reimbursement rate could not be determined. Indeed, a French policy called *Affection Longue Durée 31* (ALD31) exists specifically for serious progressive or disabling illnesses whose treatment is particularly costly and is predicted to last more than 6 months. In this case, the referring physician can ask the national health insurance scheme to fully reimburse all the care and treatments related to the patient’s illness. Hence, all orphan drugs can theoretically be reimbursed up to 100% under an ALD31. From the French patients’ point of view, the access to orphan drugs is unlikely to be hampered by financial limitations.

According to our study, 21.7% of orphan drug–therapeutic indication pairs were not marketed in France. For 72% of these pairs (18/25), a link can be established between the lack of drug availability and health technology assessment process (delays or results). First because access to drug reimbursement is needed for marketing: a negative HAS opinion followed by a negative ministerial decision for reimbursement always result in no marketing of the orphan drug. Hence, the first step for patient access is demonstrating a clinical benefit, which is a mandatory criteria for reimbursement in France. On one hand, clinical data for orphan drugs may be limited, making it difficult to assess the “value” of these drugs [20–22]. On the other hand, the severity of the disease and the lack of satisfactory therapeutic alternatives are elements in favor of orphan drugs. The relative contribution of the drug’s role in the therapeutic strategy within the clinical benefit assessment has evolved: considered as low in 2003 [23], it became a determining factor in 2014 [24]. For the 4 orphan drug-therapeutic indications pair concerned in this study, the HAS justified the insufficient actual benefit by a level of evidence considered as too low [25–28].

Then, the second impact of health technology assessment on drug availability is time-based: our study was cross-sectional, but assessment and price negotiation delays must be considered. And lastly, as marketing is a function of business, strategic and commercial choices, pharmaceutical companies can decide not to market a drug if the negotiated prices are not acceptable to them [29]. In general, high levels of clinical added value contribute to better pricing. Orphan drugs are more likely to obtain major or important clinical added value: this proportion is 19.1% in this study, but reaches 52% of orphan drugs between 2001 and 2009, versus 32% of all drugs evaluated during this period [30]. Moreover, payers value rarity in pricing decisions [31]. Between 2000 and 2010, the annual cost per patient treated per orphan drug ranged from €1251 to €407,631 (Euro zone + United Kingdom); the median cost was €32,242 [32]. In our study, most orphan drug–therapeutic indication pairs available to outpatients were fully reimbursed since these orphan drugs were judged irreplaceable and particularly expensive treatments. In France, patients do not seem to be limited by the price of orphan drugs. But the growing budgetary impact of orphan drugs is worrisome from a societal point of view [32, 33].

Drugs claiming a major, important or moderate clinical added value (i.e. 38/94 orphan drug–therapeutic indication pairs in our study) must undergo a health economic assessment if they are expected to have a significant impact on national health insurance expenditures (i.e. annual sales of €20,000,000 or more [34]). We found that 12 orphan drug–therapeutic indication pairs were slated for a health economic assessment. As a reference, 21 and 23 drugs qualified for a health economic assessment in 2014 and 2015, respectively, all drugs pooled. Major methodological limitations appeared in two efficiency opinions out of the three adopted for orphan drugs. The incremental cost-effectiveness ratio exceeded €230,000/QALY in the only published efficiency opinion for orphan drugs at the time of our study [35]. A number of researches have suggested that the commonly used health technology assessment methods are poorly suited to rare diseases: it is well known that orphan drugs are usually cost-ineffective, yet restrictions in funding may not be in line with societal preferences [36, 37]. Other studies aimed to establish societal preferences for funding drugs used to treat orphan diseases, but they have limitations and their results do not show a clear societal preference for prioritizing the treatment of rare diseases [38, 39]. Moreover, equity and ethical questions could influence decision makers [40]. Relationships between level of evidence, effect size, economic logic, ethics and equity are difficult to grasp [41].

France, like many countries, has implemented legislation to support the market access for orphan drugs [42, 43]. However, regulation is essential to ensure the sustainability of orphan drug coverage [44, 45], both at the European level and the Member State level. Since this status provides many development and financial incentives, such as specific market exclusivity, some fear that orphan drugs will become a “good business opportunity” for industry [46]. At the European level, facing the growing number of orphan drugs approved [10], the EMA has started publishing orphan maintenance assessment reports in January 2018 [47]. The objective is to assess whether a drug fulfils the criteria for orphan designation at the time of the MA to reconfirm the eligibility of the status. At the Member State level, criteria chosen for health technology assessments, combined with setting up of specific policies on orphan drugs, resulted in heterogeneity between European countries in patient accessibility to these drugs [48]. Several potential solutions exist to cover the gap between European drug market access and national patient access to licensed drugs. These include early contact between the pharmaceutical companies and the health technology assessment agencies and collaboration between the EMA and the national health technologies assessment agencies [49, 50]. In 2016, the European Working Group for Value Assessment and Funding Processes in Rare Diseases (ORPH-VAL) developed recommendations to improve the consistency of orphan drugs pricing and reimbursement assessment in Europe [51], which should optimize efficient patient access to orphan drugs in Europe.

## Conclusion

In conclusion, access to reimbursement and prices negotiation have a strong impact on effective patient access to orphan drugs in France. Prescribing restrictions and drug delivery systems influence the ease of patient supply for reimbursed orphan drugs. Patient access to orphan drugs remains incomplete and raises many questions. What is the collective interest of implementing incentives to develop and register these drugs if they are not accessible to all patients?

On one hand, this overview revealed difficulties in evaluating the reimbursement and pricing of orphan drugs. More transparency is needed to reduce the uncertainty for pharmaceutical companies about access to orphan drug reimbursement [52] to maintain their interest in rare and serious conditions. On the other hand, the orphan drug policy was marked by years without incentives followed by years with strong incentives. We believe the time has come to find a compromise in the form of reasonable and controlled incentives.

## Abbreviations

ATU: A*utorisation Temporaire d’Utilisation* (French temporary licensing system)
EU: European Union
EURORDIS: European Organization for Rare Diseases
HAS: *Haute Autorité de Santé (*French National Authority for Health)
MA: Marketing Authorization
QALY: Quality-Adjusted Life Year
RTU: *Recommandation Temporaire d’Utilisation*
